# Comparative Lipophilic Phytochemical Profiling and Bioactivity Assessment of Different Organs of *Mahonia aquifolium* (Pursh) Nutt.: Integrated Analysis of *n*-Hexane Extracts, Essential Oils and Fatty Acids

**DOI:** 10.3390/plants15172574

**Published:** 2026-08-24

**Authors:** Kübra Öğüt, Ana Belen Cerezo Lopez, Gülmira Özek

**Affiliations:** 1 Department of Pharmacognosy, Faculty of Pharmacy, Anadolu University, Eskişehir 26470, Türkiye; kubraogut@anadolu.edu.tr; 2 Departamento de Nutrición y Bromatología, Toxicología y Medicina Legal, Facultad de Farmacia, Universidad de Sevilla, C/P García González No. 2, 41012 Sevilla, Spain; acerezo@us.es

**Keywords:** *Mahonia aquifolium*, lipophilic extracts, MSD-SPME, fatty acid profiling, α-amylase inhibition, antioxidant activity

## Abstract

*Mahonia aquifolium* (Pursh) Nutt. is a medicinal plant known for its diverse phytochemical composition; however, information on the lipophilic constituents of its different organs remains limited. This study aimed to characterize the lipophilic composition and associated bioactivities of *n*-hexane extracts obtained by ultrasound-assisted extraction from leaves, flowers, fruit pulp, and seeds of *M. aquifolium*. The extracts were analyzed using GC–FID/MS of silylated extracts, FAME–GC, MSD–SPME, and UHPLC–HRMS/MS, and were evaluated for antioxidant and α-amylase inhibitory activities. Nonacosan-10-ol was identified as the predominant constituent in fruit pulp and flower extracts, whereas α-linolenic acid was the major fatty acid in leaves and seeds. Chlorogenic acid was detected as the major phenolic compound in all *n*-hexane extracts, and 3-hydroxytyrosol was also identified in the lipophilic extracts. The flower extract exhibited notable α-amylase inhibitory activity together with the highest CUPRAC and TEAC values, while the seed extract showed the strongest DPPH radical scavenging activity. The leaf extract demonstrated the highest ORAC value and β-carotene bleaching inhibition. These findings reveal substantial differences in the lipophilic composition and bioactivity profiles among *M. aquifolium* organs and provide new insights into the valorization of different plant parts as sources of bioactive lipophilic constituents.

## 1. Introduction

The sustainable utilisation of plant-derived biomass and underexploited plant fractions has become a central theme in contemporary food science and bioresource management [1,2,3]. Valorisation of secondary plant organs such as fruit pulp and seeds offers opportunities for waste reduction, circular bioeconomy development, and the generation of value-added functional ingredients [4,5]. Medicinal plants are recognized as significant sources of bioactive compounds used for the prevention and treatment of various human ailments. The Berberidaceae family, a well-established family of medicinal plants, has attracted considerable scientific interest [6]. The genus *Mahonia* Nutt., which has more than 70 species, is the second largest genus in the Berberidaceae family. *Mahonia* species are indigenous to Eastern Asia, North America, and Central America [7]. *Mahonia aquifolium* (Pursh) Nutt. is a perennial, woody shrub that is known for its invasive growth and bright yellow, fragrant flowers that bloom in April. It is also popular as an ornamental shrub since its leaves stay green all year and its flowers are stunning. Its purple fruits, commonly referred to as Oregon grapes, have further contributed to its recognition. *M. aquifolium* is a plant that comes from western North America. It has been able to grow in many different places in the Americas, Australia, and Europe, where it is grown for both decorative and medicinal purposes. In Türkiye, *Mahonia* species, notably *M. aquifolium*, are commonly cultivated in parks and gardens, particularly in humid regions [8]. Various *Mahonia* species are widely used in traditional Chinese medicine, with *M. bealei* and *M. fortunei* used for detoxification, heat clearance, and the treatment of inflammatory or infectious ailments [9,10]. In addition to their conventional applications, *Mahonia* species have been traditionally employed for the treatment of dermatological, gastrointestinal, and respiratory conditions [11]. Despite the documented pharmacological significance, the majority of research has concentrated on polar alkaloids, whereas lipophilic constituents have been relatively less extensively investigated. In parallel, research on plant secondary metabolites has progressed rapidly, particularly with the development of biotechnological approaches aimed at enhancing the production of medicinal plants rich in bioactive compounds [12,13]. Finding target metabolites for metabolic engineering, conservation efforts, and long-term farming methods still relies heavily on preliminary chemical screening. Such investigations also provide valuable reference datasets for biodiversity conservation and for guiding future biotechnological applications [14]. In this context, creating a thorough phytochemical profile of *M. aquifolium* is a significant first step.

Phytochemical analyses of the leaves, roots, and stem barks have indicated that alkaloids are the primary bioactive compounds of *Mahonia* genus. Isoquinoline alkaloids, notably protoberberines, bisbenzylisoquinolines, and aporphines, are predominant, accompanied by several secondary metabolites, including sterols, benzoquinones, and lignans [8]. Although the essential oils of *Mahonia* species have not been extensively investigated, available experimental studies have reported diverse biological activities, including analgesic, anti-inflammatory, antibacterial, antimutagenic, antiproliferative, antiviral, anticancer, and lipoxygenase inhibitory effects [9,15]. However, these findings are primarily based on in vitro and preclinical studies, and further investigations are needed to clarify their potential pharmaceutical applications. Recent studies have primarily focused on complete crude methanolic extracts or selected plant organs, most notably the roots and stem bark. In contrast, comprehensive phytochemical characterization of apolar extracts—particularly those derived from fruits, seeds, leaves, and flowers—remains limited in the available literature. Although previous phytochemical investigations on *M. aquifolium* have examined individual plant organs such as leaves, stem bark, roots, or fruits, comparative evaluation of multiple aerial and reproductive organs within the same experimental framework remains limited [6,9,10,15]. Recent studies have further demonstrated that the fruit pulp and seeds of *M. aquifolium* differ markedly in their phytochemical composition, including ascorbic acid, carotenoids, flavonoids, berberine, polysaccharides, pectins, and lipid constituents, highlighting the importance of evaluating these tissues separately rather than considering the fruit as a single entity [16]. In addition, previous investigations have shown that different organs have been shown to differ in their phytochemical composition and biological properties, reflecting their specialized physiological functions and metabolic composition [8]. Therefore, in the present study, the fruit pulp and seeds were evaluated separately together with leaves and flowers to obtain a more comprehensive understanding of organ-specific lipophilic metabolite distribution and its relationship with the observed biological activities. Such an approach facilitates the identification of plant organs with greater potential as sources of bioactive lipophilic constituents for pharmaceutical, nutraceutical, and functional food applications, while also supporting the selective valorisation of different plant fractions and the sustainable utilization of plant biomass. Furthermore, investigating the same plant organs using a lipophilic extraction strategy enables a more comprehensive understanding of organ-dependent metabolite distribution and complements previous studies based on polar extracts, thereby providing a broader phytochemical characterization of *M. aquifolium* [8]. Considering the documented pharmacological importance of *M. aquifolium* alkaloids and the nutritional value of its fruits and seeds [8,9,10,15], comprehensive organ-specific characterization of lipophilic constituents may facilitate the rational selection of plant materials for future pharmaceutical, nutraceutical, and functional food applications. The analytical strategy employed in this study aims to overcome key limitations of conventional phytochemical workflows, which frequently rely on single-organ extraction using polar solvents and may therefore overlook volatile, esterified, or lipophilic constituents [17]. By deploying solvent-partitioning, silylation, and headspace enrichment techniques in an organ-resolved manner, the present work offers enhanced compositional depth and chemotaxonomic resolution [18,19].

In addition to the analytical platforms employed, ultrasound-assisted extraction (UAE) was used for the preparation of *n*-hexane extracts. UAE is a widely applied extraction technique in natural product research because it enhances mass transfer through acoustic cavitation, thereby improving solvent penetration into plant tissues and facilitating the efficient recovery of target compounds. Compared with conventional extraction methods, UAE reduces extraction time and solvent consumption while minimizing thermal degradation of thermolabile constituents. Owing to these advantages, UAE has become an effective and environmentally friendly approach for the extraction of bioactive metabolites from plant materials [20,21,22,23,24]. In the present study, UAE was employed to obtain lipophilic *n*-hexane extracts for subsequent phytochemical characterization and biological activity evaluation.

Considering the physicochemical diversity of volatile and semi-volatile constituents, this study applies a dual extraction strategy combining hydrodistillation (HD) and microsteam distillation–solid phase microextraction (MSD-SPME) to ensure broad coverage of aroma-relevant compounds [25,26]. HD is commonly used to separate essential oils on a large scale, and it can recover less volatile, high-boiling compounds. However, the application of HD may lead to thermal degradation or loss of heat-sensitive volatile compounds [27,28,29]. In contrast, MSD-SPME operates under mild conditions, requires minimal sample preparation, and is recognized as a green analytical method due to its solvent-free, rapid, and low-energy nature [30,31]. MSD-SPME is especially efficient at identifying compounds that are exceedingly volatile and thermolabile, which could be missed in traditional distillation procedures [32,33]. The combined use of HD and MSD-SPME produces a wider and more accurate representation of the volatile compounds in the plant organs that were investigated. Previous research has demonstrated the efficacy of analogous dual-approach methods [34,35,36]. Additionally, silylation has been applied to improve the volatility and thermal stability of non-volatile and semi-volatile compounds—especially phenolic acids and triterpenoids—facilitating their effective gas chromatography–mass spectrometry (GC-MS) analysis as trimethylsilyl (TMS) derivatives [37,38,39]. Collectively, these analytical strategies enable a comprehensive characterization of the apolar phytochemical composition of the aerial and reproductive parts of *M. aquifolium*.

From an analytical perspective, the combination of GC-MS (after silylation), fatty acid methyl ester gas chromatography (FAME-GC), and microsteam distillation–solid-phase microextraction (MSD-SPME) within the same matrix enables comprehensive characterization of volatile and semi-volatile lipophilic metabolites, illustrating the synergistic capabilities of these platforms for plant matrix deconvolution. The current work is geared toward thoroughly profiling the lipophilic phytochemical profiles of essential oils and *n*-hexane extracts derived from the leaves, flowers, fruit pulp, and seeds of *M. aquifolium*, as well as assessing their biological activities. The work offers valuable insights into the bioactive potential of the plant’s lipophilic constituents by combining chemical composition analysis with antioxidant and enzyme inhibition assays.

Despite substantial research on the polar alkaloid content of *Mahonia* species, the comparative analysis of lipophilic extracts from various plant organs—particularly with gas chromatography coupled with flame ionization detection and mass spectrometry (GC-FID/MS) after silylation, FAME-GC, and MSD-SPME—remains limited. To the best of our knowledge, no previous study has concurrently applied silylation-assisted GC–FID/MS, FAME–GC, and MSD-SPME approaches to characterize the lipophilic metabolome of *M. aquifolium* across multiple aerial and reproductive organs. This integrative analytical strategy, combined with UHPLC–MS/MS (ultra-high-performance liquid chromatography–tandem mass spectrometry), enables a comprehensive comparative assessment of apolar constituents within the genus *Mahonia*. Overall, this study aims to evaluate organ-specific lipophilic profiles in the context of sustainable plant resource utilisation and to assess their potential as functional bioactive ingredients.

## 2. Results and Discussion

### 2.1. Extraction Procedures

#### 2.1.1. Extraction of Plant Material

Comparative phytochemical and bioactivity analyses were performed on lipophilic *n*-hexane extracts prepared from the flowers, leaves, fruit pulp, and fruit seeds of *M. aquifolium* using ultrasound-assisted extraction (UAE). Considerable differences in extraction yield were observed among the investigated organs. Fruit seeds produced the highest amount of apolar extract (10.7 g), corresponding to 26.76% of the air-dried plant material. In contrast, flower, leaf, and fruit pulp extracts yielded 1.59 g (3.99%), 3.32 g (2.08%), and 0.53 g (1.33%), respectively. Essential oils were obtained in substantially lower amounts, with yields of 0.02 g (0.05%) and 0.01 g (0.03%) from flowers and leaves, respectively. The results revealed substantial differences in extraction yield among the investigated plant organs, particularly in fruit seeds, which exhibited considerably higher recovery of lipophilic extracts than the aerial parts. The markedly higher yield obtained from fruit seeds suggests that this organ represents a concentrated source of apolar metabolites, including fatty acids and other lipophilic constituents [40,41]. From a sustainable resource utilization perspective, seeds and fruit-derived materials are frequently underutilized despite their phytochemical potential [4,42]. The efficient recovery of lipophilic constituents by ultrasound-assisted *n*-hexane extraction highlights the potential of different *M. aquifolium* organs as valuable sources of bioactive compounds and supports their selective valorization within circular bioeconomy and sustainable biomass utilization strategies [43,44].

#### 2.1.2. Comparative Analysis of Volatile Constituents and Fatty Acids by MSD-SPME, Hydrodistillation, and GC-FID/MS

**Figure 1:**
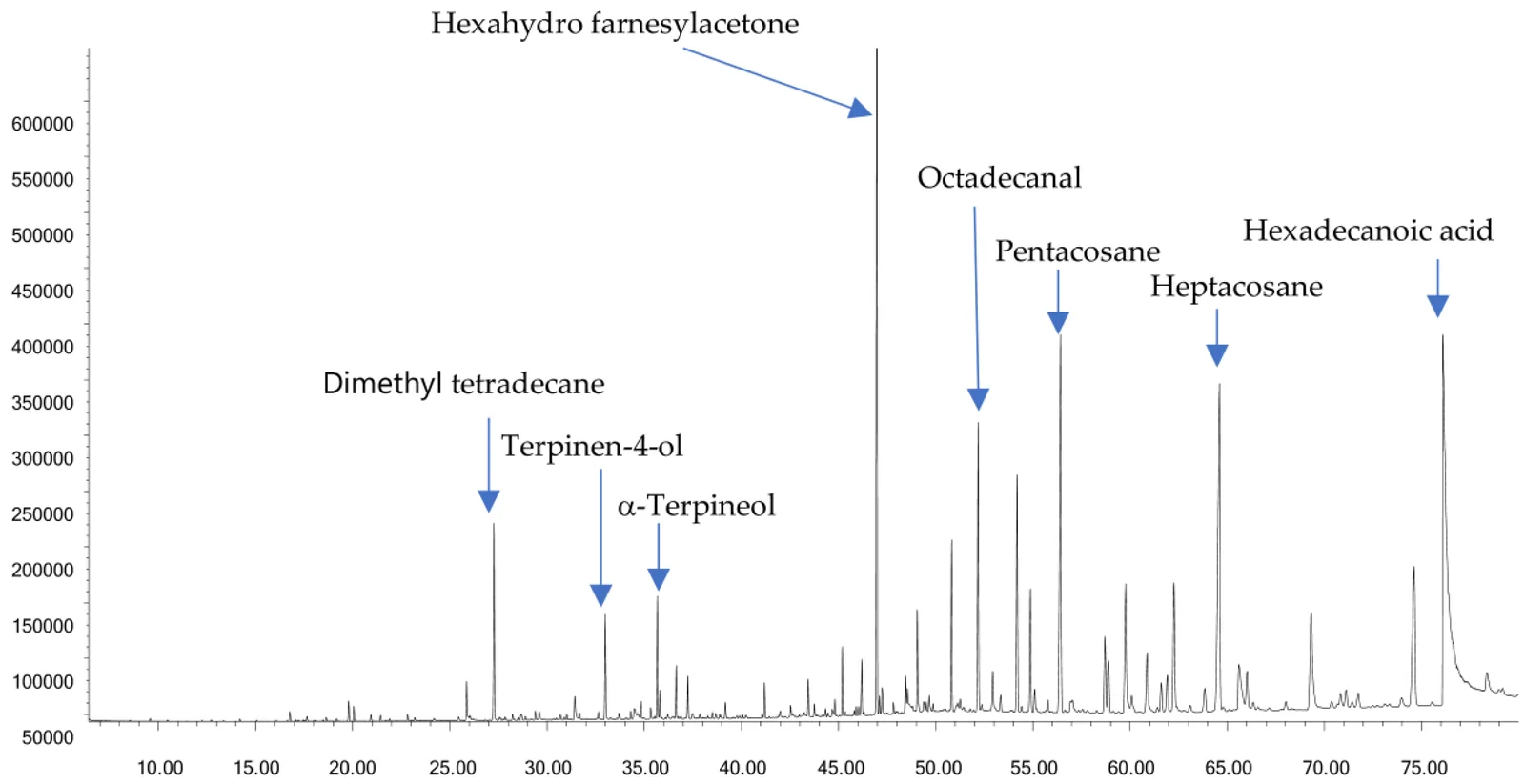
Representative GC-FID chromatogram of the essential oil obtained from *M. aquifolium* flowers. Only the major volatile constituents are labeled in the chromatogram, while the complete list of identified compounds is provided in Supplementary Material S1.

**Figure 2:**
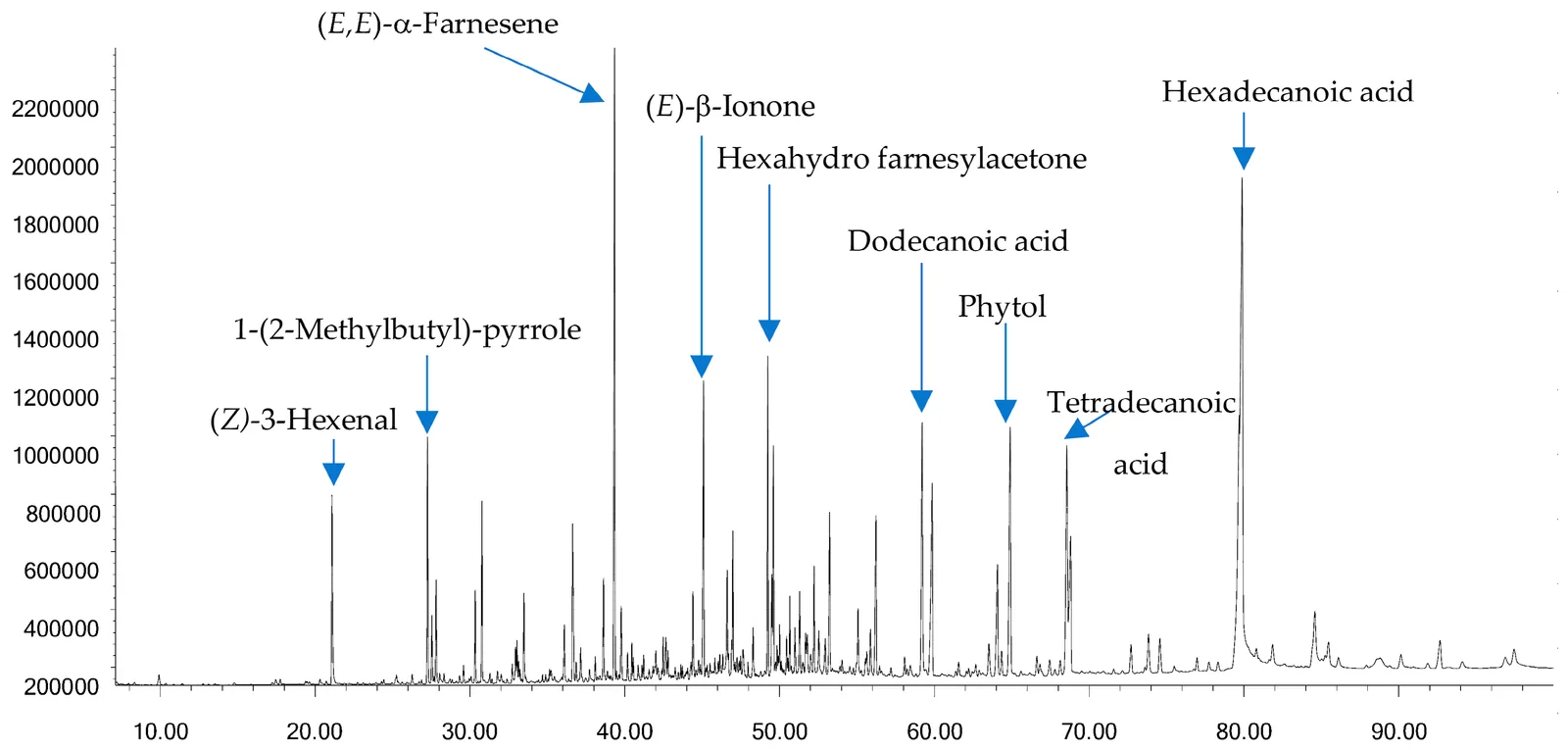
Representative GC-FID chromatogram of the essential oil obtained from *M. aquifolium* leaves. Only the major volatile constituents are labeled in the chromatogram, while the complete list of identified compounds is provided in Supplementary Material S1.

Gas chromatographic analysis of the *M. aquifolium* flower essential oil led to the identification of 43 compounds, accounting for 99.67% of the total oil composition. The fatty acids, alkanes and sesquiterpenes were found to be the major constituents. Namely, hexadecanoic acid (19.40 ± 0.03%), heptacosane (11.89 ± 0.01%), and hexahydrofarnesyl acetone (10.73 ± 0.01%) were identified as the major constituents of the flower essential oil obtained via hydrodistillation (Figure 1). Similarly, the leaf essential oil contained 136 compounds, representing 92.79% of the total oil. The principal components of the leaf essential oil were fatty acids, hexadecanoic acid (18.78 ± 0.18%), and tetradecanoic acid (4.10 ± 0.01%). (*E*,*E*)-α-Farnesene (5.70 ± 0.08%) was found as the major sesquiterpene in the leaf oil (Figure 2). The complete composition of both essential oils is provided in Supplementary Material S1.

**Figure 3:**
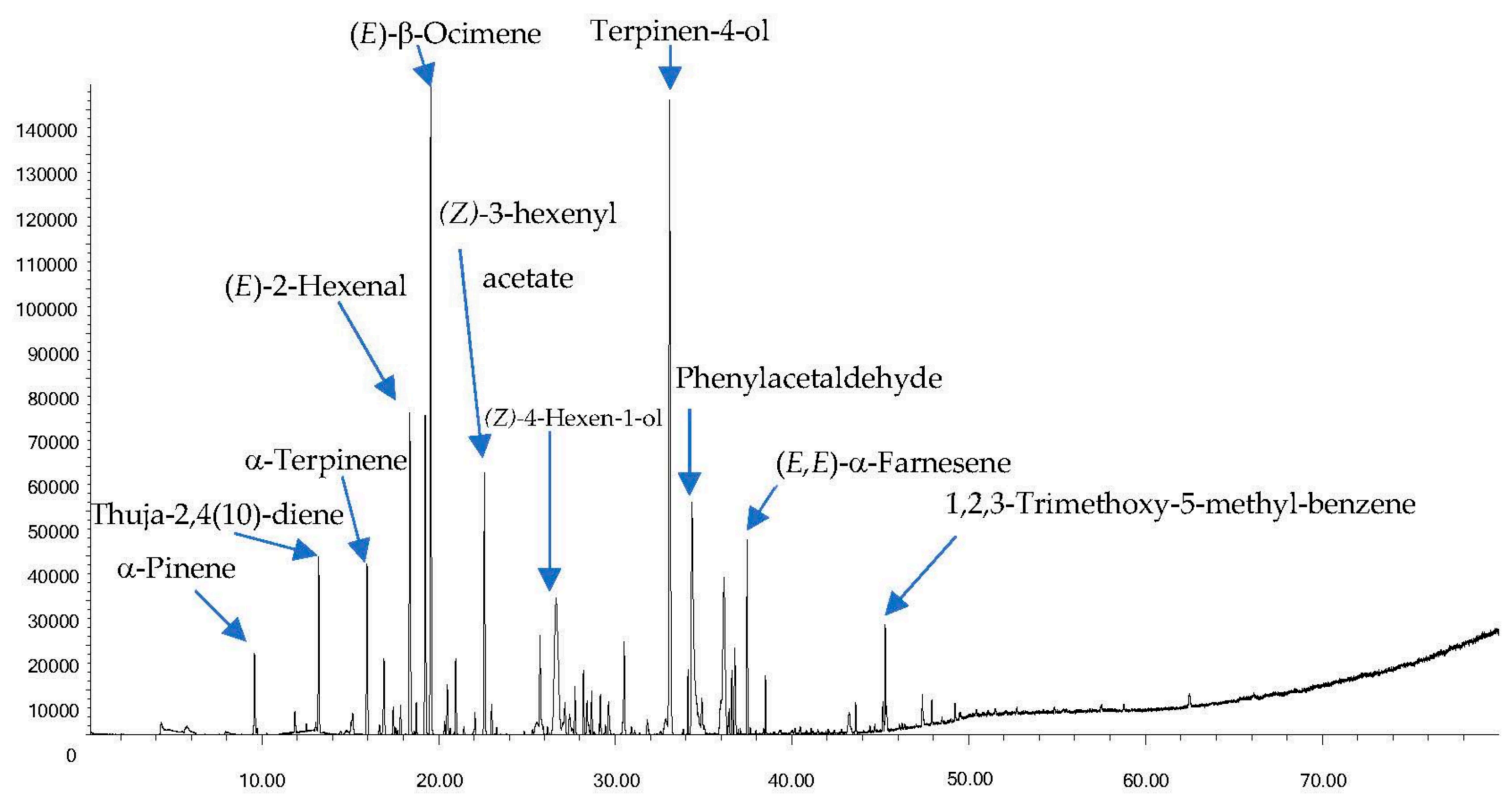
MSD-SPME–GC–MS chromatogram of the major volatile constituents from *M. aquifolium* flowers. All identified compounds are presented in Supplementary Material S1.

**Figure 4:**
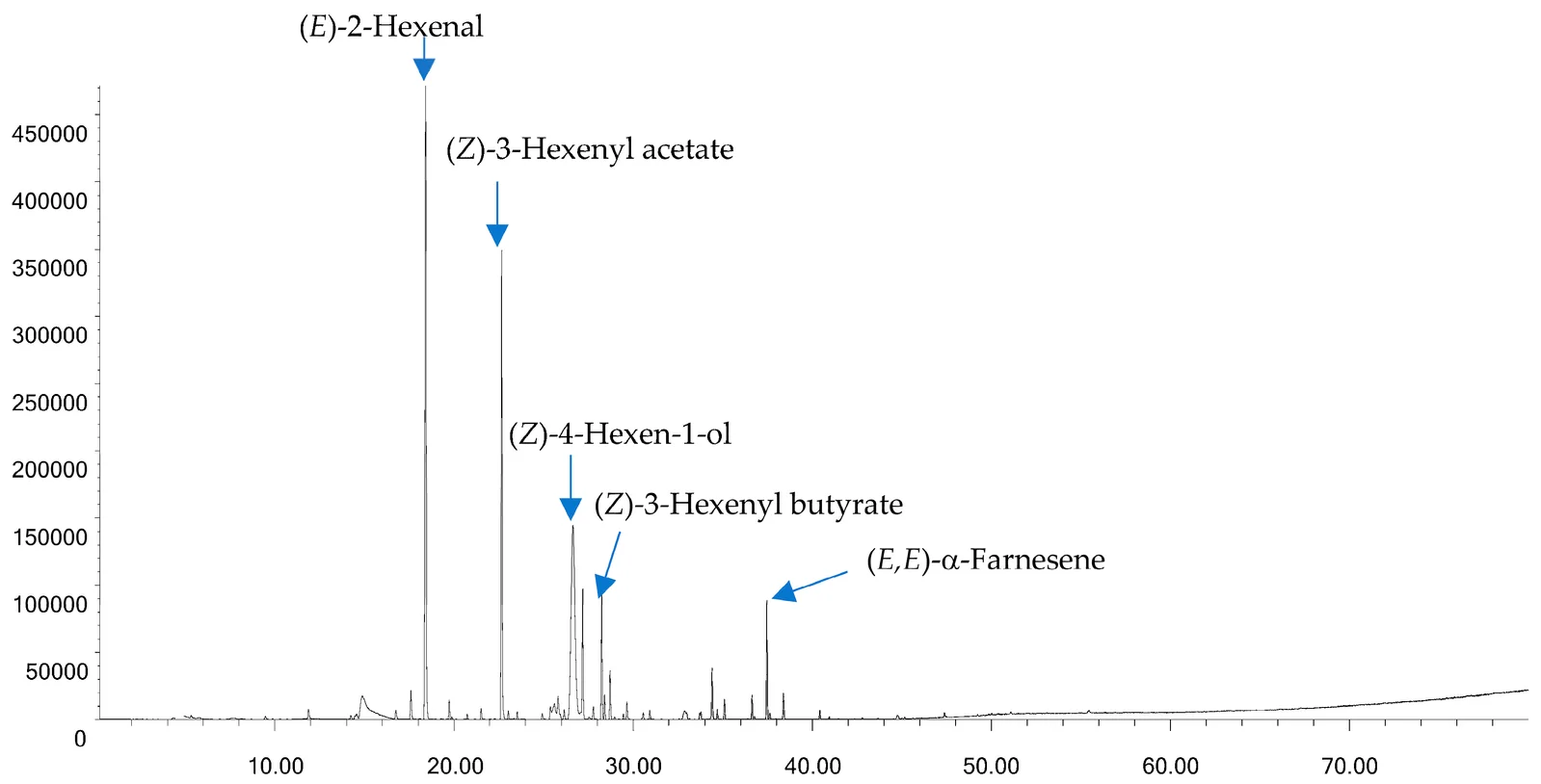
MSD-SPME–GC–MS chromatogram of the major volatile constituents from *M. aquifolium* leaves. All identified compounds are presented in Supplementary Material S1.

Analysis of the volatiles from *M. aquifolium* leaves and flowers using the MSD-SPME method revealed 39 and 49 volatile components, accounting for 98.43% and 99.08% of the total volatiles detected, respectively. The major volatile constituents identified in the flowers were terpinen-4-ol (14.79 ± 0.19%), (*E*)-β-ocimene (11.19 ± 0.11%), and benzeneacetaldehyde (9.79 ± 0.12%) (Figure 3). In the leaves, (*E*)-2-Hexenal (27.03 ± 0.10%) and (*Z*)-4-hexen-1-ol (26.29 ± 0.10%) were determined as the principal volatile compounds (Figure 4). The full volatile constituents’ lists are presented in Supplementary Material S1. The volatile compositions identified in the present study were compared with those previously reported for other *Mahonia* species to evaluate similarities and species-specific differences. Gas chromatographic analysis of *M. bealei* (Fortune) Carr leaf essential oil led to the identification of 26 compounds, accounting for 70.86% of the oil. The main constituents were ethyl linoleate, 2,6,10,14-tetramethyl octadecane, and tetracosane [45]. Similarly, the essential oil from *M. retinervis* leaves, representing 64.59% of the total oil, was primarily composed of tetracosane and phytol [46]. In *M. jingxiensis* leaves, phytol isomers, eicosane, and heptacosane were the dominant compounds, comprising 69.94% of the essential oil [47]. The essential oil of *M. fortunei* constituted 60.72% of the total oil content, with 2,6,10,14-tetramethyl octadecane, ethyl linoleate, and tetracosane identified as the principal components [46,48]. Furthermore, the essential oil of *M. duclouxiana* made up 75.65% of the oil, with tetracosane, 2,6,10,14-tetramethyl octadecane, and ethyl linoleate being the major constituents [47]. In addition, the volatile profile of *M. japonica* was reported to be rich in (*E*)*-*β-ocimene (57.0%), benzyl alcohol (14.0%), and citronellol (6.8%) [49]. Hexadecanoic acid (54.49%) and linoleic acid (5.98%) were identified as the predominant compounds in the stems and leaves of *M. bodinieri* [50]. Additionally, a study on *M. breviracema* cultivated under different light environments revealed that the leaf essential oils were particularly rich in hexadecanoic acid (ranging from 10.54% to 72.19%) and α-ionone (1.25–42.39%), demonstrating how environmental conditions may significantly influence volatile oil composition [47].

Various factors, including species, developmental stages, and environmental conditions, influence the chemical composition of essential oils [51]. In this study, a comparative analysis of the volatile compounds in the leaves and flowers of *M. aquifolium* revealed that they do not share the same major constituents. Moreover, the chemical composition of *M. aquifolium* was found to differ significantly from that of other *Mahonia* species.

**Figure 5:**
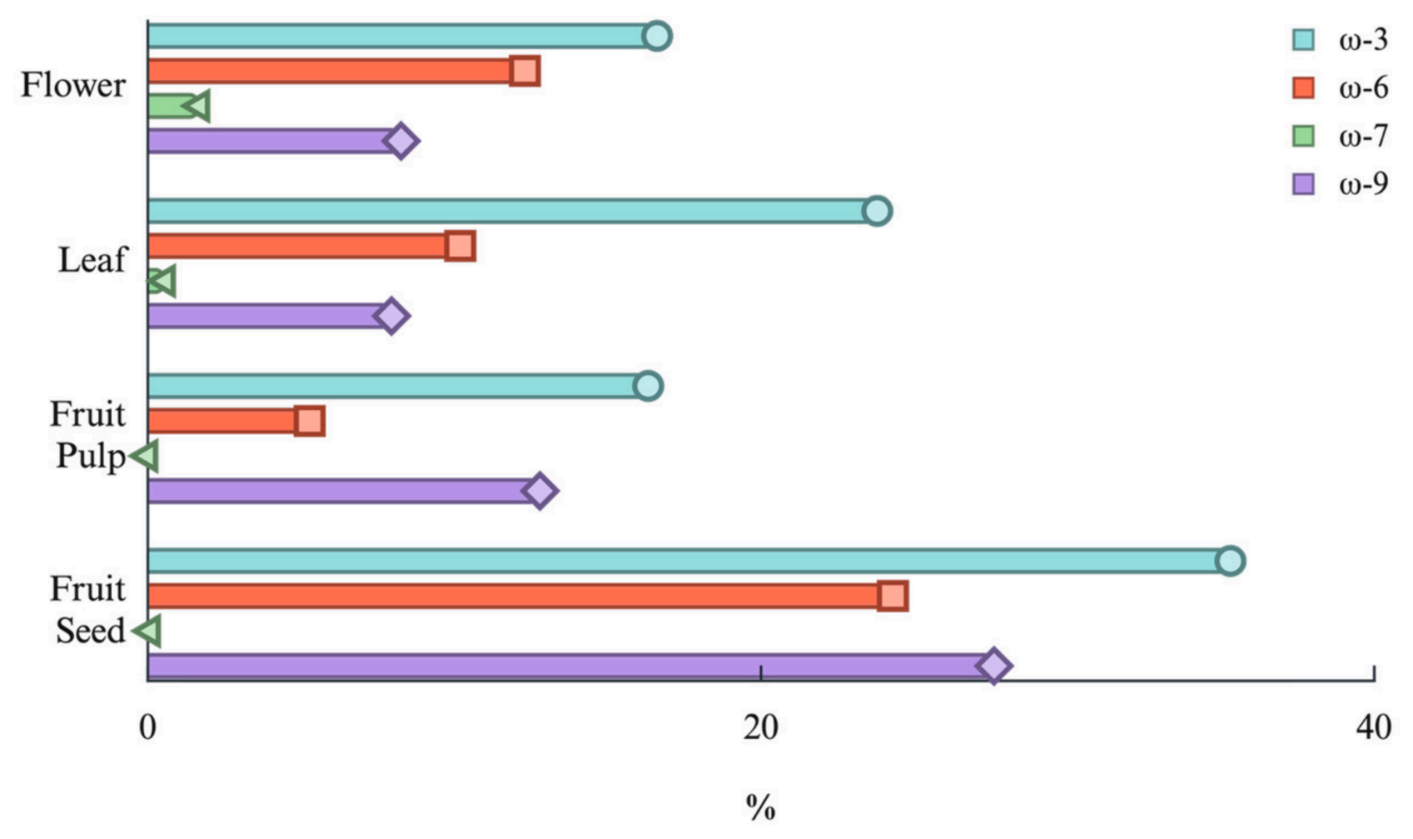
Distribution of total ω-3, ω-6, ω-7, and ω-9 fatty acids as percentages of total fatty acids in the flowers, leaves, fruit pulp, and fruit seeds of *M. aquifolium*. A detailed breakdown of the individual fatty acids is available in Supplementary Material S1.

Fatty acids are categorized into two primary groups—saturated and unsaturated—based on their chemical structure. Plants contain three primary classes of unsaturated fatty acids: omega-3, omega-6, and omega-9. Omega-6 and omega-3 are essential fatty acids that play a critical role in human nutrition. The body can synthesize omega-9 fatty acids from other fatty acids, making them non-essential [52]. The fatty acid composition of different parts of *M. aquifolium*—including flowers, leaves, fruit pulp, and fruit seeds—was comprehensively evaluated (Figure 5). The dominant ω-3, ω-6, and ω-9 fatty acids varied significantly among the plant parts. Notably, ω-3 content was highest in the fruit seeds, while flowers and leaves also showed considerable levels. This distribution pattern underscores the potential of specific plant organs, particularly seeds, as promising sources of essential fatty acids within nutrition-oriented and sustainable food system frameworks. Detailed fatty acid profiles for each part are provided in Supplementary Material S1.

In the present work, we have identified a total of 16 fatty acids, accounting for 97.79% of the fatty acids present in *M. aquifolium* flowers. The major fatty acids identified were palmitic acid (22.47 ± 0.18%), linolenic acid (16.61 ± 0.09%), and linoleic acid (16.6%). *M. aquifolium*’s leaves comprised a total of 14 fatty acids, representing 93.00% of the composition. The major fatty acids identified in the leaves were linolenic acid at 23.79 ± 0.14% and palmitic acid at 22.24 ± 0.26%. The pulp oil of *M. aquifolium* comprised 7 fatty acids. The primary fatty acids identified were palmitic acid (41.60 ± 0.17%) and nonadecanoic acid (19.79 ± 0.11%). *M. aquifolium*’s fruit seeds comprise a total of 13 fatty acids. The primary fatty acids in the seeds were linolenic acid, comprising 35.3%, and oleic acid, accounting for 26.37 ± 0.15%.

In a previous study focusing solely on *M. aquifolium* seed oil, α-linolenic acid (C18:3n3c) was identified as the dominant fatty acid, comprising 46.67 ± 0.25% of the total fatty acid content, followed by linoleic acid (C18:2n6c, 25.13 ± 0.15%) and oleic acid (C18:1n9c, 20.07 ± 0.24%). Saturated fatty acids (SFAs) made up only 8.12%, while polyunsaturated fatty acids (PUFAs) were predominant at 71.80%, indicating a high degree of unsaturation and potential nutritional value [8].

In contrast, our study provides a broader fatty acid profile not only for the seed oil but also for the leaves, flowers, and fruit pulp of *M. aquifolium*. While the seed extract in our analysis was also rich in linolenic acid (35.31 ± 0.16%) and oleic acid (26.37 ± 0.15%), the flower and leaf extracts showed varying dominant components such as palmitic acid and linolenic acid, underlining organ-specific variation in lipophilic composition. This broader evaluation emphasizes the distinct fatty acid distribution across plant parts, which had not been reported in earlier studies focusing solely on the seed oil.

The pronounced organ-dependent variation in fatty acid composition observed in the present study has important implications from a sustainable food utilisation perspective. The substantial levels of polyunsaturated fatty acids (PUFAs), including ω-3 and ω-6, together with notable proportions of ω-9 fatty acids across different plant organs, highlight their nutritional relevance. In particular, the seed material exhibited a rich unsaturated fatty acid profile, while leaves and pulp demonstrated distinct enrichment patterns. Considering that fruit pulp and seeds are frequently underexploited in ornamental cultivation systems, their compositional richness supports the development of value-added functional ingredients and contributes to sustainable biomass utilisation strategies.

#### 2.1.3. Chemical Composition of Lipophilic Extracts

The lipophilic constituents extracted with *n*-hexane from *M. aquifolium* have previously been derivatized with N,O-bis(trimethylsilyl)trifluoroacetamide (BSTFA) reagent to get silyl derivatives for subsequent gas chromatographic analysis. Chromatographic resolution of the volatile silyl derivatives was achieved by applying the temperature program optimization on an apolar column (HP-5). *n*-Alkanes (C_8_–C_40_) were used as reference points in the calculation of relative retention indices (RRIs). Quantification of volatile components was performed on the basis of their GC/FID peak areas using integration data without normalization. The list of the identified compounds is presented in Table 1.

Silylation is a widely used derivatization technique that enables the analysis of compounds containing polar functional groups by the gas chromatographic method. This process specifically transforms compounds containing low-volatility functional groups such as hydroxyl (-OH), carboxyl (-COOH), and amino (-NH_2_) into more volatile and thermally stable derivatives. Through silylated derivatives, GC-FID/MS analysis allows for sharper peak resolution and more reliable quantitative results, while also facilitating the analysis of polar matrices. Although silylation is primarily used for the analysis of polar compounds, its application has been extended to include certain slightly apolar structures such as long-chain alcohols and hydroxylated fatty acids [53]. Thus, both polar and select apolar compounds can be simultaneously evaluated within the same analytical platform. In this respect, silylation offers a significant advantage for the comprehensive characterization of complex phytochemical mixtures [54].

GC-FID/MS analysis of *M. aquifolium n*-hexane extracts revealed nonacosan-10-ol as the predominant apolar compound in the leaves (10.04 ± 0.01%), flowers (21.10 ± 0.11%), and fruit pulp (38.43 ± 0.15%). In the seed extract, (*Z*)-9-octadecenoic acid (oleic acid) was identified as the major constituent (23.40 ± 1.58%).

To the best of our knowledge, this is the first study to report the GC-FID/MS analysis of the silylated apolar (*n*-hexane) extracts in the genus *Mahonia*. A comparison with our previously published study on the silylated polar extracts of *M. aquifolium* further demonstrates the strong influence of extraction solvent polarity on the recovered metabolite profile. In the polar extracts, sucrose (34.18%) and crypto-chlorogenic acid (27.94%) were the predominant constituents in leaves, and crypto-chlorogenic acid (23.52%) dominated the flower extract, while malic acid (17.50%) and β-D-glucopyranose (13.42%) were the major metabolites in fruit pulp [8]. In contrast, the present *n*-hexane extracts were characterized by the predominance of nonacosan-10-ol in leaves, flowers, and fruit pulp, whereas oleic acid was the major constituent of the seed extract. These findings clearly indicate that solvent polarity substantially affects the classes of metabolites recovered, with polar extraction favoring carbohydrates and phenolic acids, while *n*-hexane extraction preferentially enriches long-chain alcohols, fatty acids, and other lipophilic constituents [55,56]. Together, these complementary approaches provide a more comprehensive understanding of the phytochemical diversity of *M. aquifolium*. The absence of such data for any *Mahonia* species in the literature highlights the novelty and originality of the present findings. Additionally, the predominance of nonacosan-10-ol in the leaves, flowers, and fruit pulp indicates a consistent enrichment of long-chain alcohols across aerial and reproductive organs. Long-chain aliphatic alcohols are known to contribute to structural and protective plant lipid matrices and may influence physicochemical properties relevant to extract stability [57,58]. In contrast, the dominance of oleic acid in the seed extract is particularly noteworthy from a nutritional standpoint, as monounsaturated fatty acids such as oleic acid are widely associated with balanced dietary lipid profiles [59,60]. The organ-specific distribution of these major constituents highlights the potential for selective utilisation of different plant extracts depending on desired functional or compositional attributes, supporting sustainable valorisation strategies.

#### 2.1.4. UHPLC-HRMS/MS Orbitrap Phenolic Compound Analysis

In our investigation, both qualitative and quantitative analyses of phenolic compounds in lipophilic (*n*-hexane) extracts of *M. aquifolium* were performed. Through UHPLC-HRMS/MS analysis, it was possible to identify and quantify the following compounds: 3-hydroxytyrosol, 4-hydroxybenzoic acid, 4-*O*-caffeoylquinic acid, apigenin-7-*O*-glucoside, caffeic acid, catechin, chlorogenic acid, p-coumaric acid, diosmetin, flavanomarein, gentisic acid, isorhamnetin, luteolin, naringin, and vanillin.

Phenolic compounds were identified and quantified by UHPLC–HRMS/MS analysis. A standard mixture of commercially available reference compounds was prepared and serially diluted into twelve concentration levels. Calibration curves were constructed for each analyte to determine phenolic concentrations in the extracts. Detailed identification and quantification data are provided in Supplementary Materials S2–S5, and the main findings are summarized in Table 2.

The present study provides a detailed comparison of individual phenolic constituents in *n*-hexane extracts obtained from different organs of *M. aquifolium*, revealing pronounced organ-dependent variation in compound distribution. Notably, chlorogenic acid was detected at remarkably high concentrations in the seed extract, while flowers and leaves also exhibited distinct phenolic enrichment patterns. These findings demonstrate that even apolar extraction systems can recover measurable levels of phenolic compounds, contributing to a more comprehensive understanding of extract composition. The detection of phenolic compounds in *n*-hexane extracts may be attributed to co-extraction effects, matrix interactions, or limited solubility of certain phenolics under the applied extraction conditions. A comparison with our previous UHPLC-HRMS/MS investigation of the 70% aqueous ethanol extracts of *M. aquifolium* further emphasizes the influence of solvent polarity on phenolic recovery. In the polar extracts, chlorogenic acid was consistently identified as the predominant phenolic constituent, reaching 786.06 mg/g_dry extract_ in flowers, while 4-hydroxybenzoic acid, 4-*O*-caffeoylquinic acid, catechin, and flavanomarein were also detected at considerable levels depending on the plant organ [8]. In contrast, the present *n*-hexane extracts contained substantially lower concentrations of phenolic compounds, although chlorogenic acid remained the dominant phenolic constituent [8]. These findings indicate that polar extraction preferentially enriches phenolic acids and flavonoids, whereas *n*-hexane extraction primarily recovers lipophilic metabolites while still allowing the co-extraction of selected phenolic compounds. Together, these complementary extraction approaches provide a broader representation of the chemical diversity of *M. aquifolium*.

From a sustainable food utilisation perspective, the identification of nutritionally relevant phenolics across multiple plant extracts highlights the potential for targeted valorisation of specific organs [61]. Such compositional diversity supports the development of value-added plant-derived ingredients and reinforces the importance of organ-resolved evaluation when considering sustainable biomass utilisation strategies [62,63].

### 2.2. Total Phenolic, and Flavonoid Contents

Total phenolic content (TPC) is widely used as an indicator of the overall reducing capacity of plant extracts and is frequently correlated with antioxidant potential. To determine the phenolic content of the extracts, phenolic capacity has been determined using Folin–Ciocalteu reagent. The outcomes were then comparatively studied and presented as the gallic acid equivalents (GAE). To determine the flavonoid content the extracts were subjected to spectrophotometric analysis using an AlCl_3_ reagent. The results of an analysis of the extracts’ flavonoid content are presented as rutin equivalents (RE).

The *n*-hexane extracts of *M. aquifolium* revealed the highest total phenolic concentration in the leaf extract (40.02 ± 0.89 mg GAE/g_extract_), followed by the flower extract (18.55 ± 1.16 mg GAE/g_extract_). The fruit pulp and seed extracts demonstrated somewhat lower phenolic concentrations, measuring 11.15 ± 0.36 and 10.21 ± 0.92 mg GAE/g_extract_, respectively. Regarding flavonoid content, only the seed extract produced a measurable result (1.22 ± 0.01 mg RE/g_extract_), whereas the other extracts did not exhibit detectable amounts under the specified conditions. Our investigation showed that the leaf *n*-hexane extract had the highest total phenolic content (40.02 ± 0.89 mg GAE/g_extract_), while the fruit seed extract was the only one with a measurable flavonoid content (1.22 ± 0.01 mg RE/g_extract_). This result aligns with the UHPLC-HRMS/MS findings presented in Table 2. In a previous study, the total phenolic content of *M. aquifolium* berry chloroform extract was reported as 1.30 ± 0.22 mg GAE/100 g fresh weight [64]. However, direct comparison with the present study is not appropriate because of differences in extraction solvent, plant material, and the basis used to express the results (fresh weight versus extract weight). Therefore, the literature data are provided only for general reference. In the present study, the total phenolic content of the *n*-hexane extracts ranged from 10.21 ± 0.92 to 40.02 ± 0.89 mg GAE/g_extract_, indicating that measurable amounts of phenolic compounds can also be recovered from apolar extracts, particularly from leaf and seed materials [65]. These findings support the potential of organ-specific apolar extraction strategies for recovering valuable bioactive constituents [65]. In a previous study on the 70% aqueous ethanol extracts of *M. aquifolium*, total phenolic contents ranged from 19.43 ± 1.32 to 100.16 ± 1.90 mg GAE/g_extract_, with the highest values recorded for the leaf and fruit seed extracts. In comparison, the present study revealed lower total phenolic contents in the *n*-hexane extracts, ranging from 10.21 ± 0.92 to 40.02 ± 0.89 mg GAE/g_extract_ [8]. These differences are consistent with the distinct extraction selectivities of polar and apolar solvents. While aqueous ethanol preferentially recovers phenolic acids and flavonoids, *n*-hexane extraction primarily targets lipophilic constituents, although measurable amounts of phenolic compounds may also be co-extracted. Although the spectrophotometric assays provided useful comparative information on the overall phenolic and flavonoid contents of the extracts, the UHPLC-HRMS/MS analysis offered a more selective and reliable characterization of individual phenolic constituents. Therefore, the chromatographic data provided the primary basis for interpreting the organ-specific phenolic composition observed in the present study. Collectively, the spectrophotometric and chromatographic approaches provided complementary phytochemical information and contributed to a more comprehensive evaluation of the chemical diversity of *M. aquifolium*.

### 2.3. Biological Activity Results

*M. aquifolium n*-hexane extracts of flowers, leaves, fruit pulps, and seeds were tested for antioxidant activity using the 2,2-diphenyl-1-picrylhydrazyl (DPPH) radical scavenging assay, oxygen radical absorbance capacity (ORAC), β-carotene bleaching inhibition, and cupric reducing antioxidant capacity (CUPRAC) assays. Furthermore, the extracts were evaluated for α-amylase inhibition to evaluate anti-diabetic effects in vitro. Results were compared to acarbose, a typical α-amylase inhibitor. The results of antioxidant activity evaluation as well as α-amylase activity are presented in Table 3.

Among the *n*-hexane extracts derived from different parts of *M. aquifolium*, distinct variations in antioxidant and enzyme inhibitory activities were observed. The leaf extract exhibited the strongest ORAC activity (0.452 ± 0.003 µmol trolox/mg_extract_) and the most effective inhibition of β-carotene peroxidation (IC_50_: 0.34 ± 0.00 mg/mL). In contrast, the flower extract showed the highest TEAC value (0.02 ± 0.00 µmol trolox/g_dry weight_) and the most potent α-amylase inhibition (IC_50_: 0.16 ± 0.00 mg/mL).

The variation in plant parts, extraction methodologies, and reporting formats in the literature restricts direct numerical comparison of antioxidant and α-amylase inhibitory effects among *Mahonia* species. Consequently, the current findings are analyzed in relation to defined experimental conditions and statistical assessment, offering an additional viewpoint to prior research mostly centered on polar extracts or isolated chemicals. Additionally, these findings underscore the phytochemical diversity and bioactivity potential among the plant’s anatomical parts and support the relevance of part-specific apolar extraction strategies for *M. aquifolium*. The observed antioxidant activity appears to be consistent with the phenolic levels detected in the extracts, reinforcing the role of phenolics in the bioactivity of lipophilic extracts of *M. aquifolium*. The significant α-amylase inhibitory activity detected in the flower *n*-hexane extract potentially correlates with the presence of several major compounds. Palmitic acid has been reported to influence enzyme-related processes in certain biological systems [66]. Furthermore, terpinen-4-ol and (*E*)*-*β-ocimene, recognized as principal volatiles using MSD-SPME analysis, have exhibited diverse biological activities, including enzyme regulation [32]. Chlorogenic acid, a polyphenolic molecule with well-documented α-amylase inhibitory characteristics, further substantiates the reported action [67]. In addition, nonacosan-10-ol-TMS, identified as a principal derivatized component, may contribute to the observed activity through hydrophobic interactions, although specific mechanistic pathways require further investigation [68]. Together, these compounds likely act synergistically to inhibit α-amylase activity.

The leaf *n*-hexane extract demonstrated the most significant antioxidant activity among all evaluated extracts, attributable to its distinctive phytochemical composition. α-Linolenic acid, a major ω-3 fatty acid, has been associated with antioxidant-related physiological effects. It is typically related to stabilizing membranes and free radical scavenging [69]. Palmitic acid, which is also included as a major compound in the essential oil of the leaves, may play a role in antioxidant defense, especially via membrane-related pathways [70]. The volatile (*Z*)-4-hexen-1-ol that MSD-SPME analysis found is widely known for its antioxidant and aroma characteristics [71]. UHPLC-HRMS/MS analysis shows that the presence of chlorogenic acid, an effective natural antioxidant, is closely associated with the observed high radical scavenging activity [67]. Furthermore, nonacosan-10-ol, which is the main part of the derivatized extract, may help with antioxidant capacity by interacting with water and protecting lipid structures [68]. These findings indicate that the antioxidant activity of the leaf extract arises from the synergistic interactions of its various lipophilic and phenolic constituents. Although the present study employed widely accepted chemical antioxidant assays, future studies evaluating antioxidant activity against biologically relevant reactive oxygen and nitrogen species or using cell-based oxidative stress models would provide further insight into the physiological relevance of these findings.

## 3. Materials and Methods

### 3.1. Chemicals

The chemicals used in this study, including *n*-hexane (≥99%), dimethyl sulfoxide (DMSO) (≥99.9%), methanol (≥99.85%), ethanol (≥99.5%), formic acid (90%), hydrochloric acid (37%), glacial acetic acid (100%), Folin–Ciocalteu reagent (FCR), boron trifluoride (BF_3_) reagent, N,O-bis(trimethylsilyl)trifluoroacetamide containing 1% trimethylchlorosilane (BSTFA, with 1% TMCS), and butylated hydroxyanisole (BHA) (≥98.5%), were of analytical grade or higher and purchased from Sigma-Aldrich (St. Louis, MO, USA). The lipid extraction kit, α-amylase from porcine pancreas (Type VI-B, ≥5 units/mg solid), and acarbose were also obtained from Sigma-Aldrich (St. Louis, MO, USA). Other chemicals of analytical grade were acquired from Merck (Darmstadt, Germany). Standard *n*-alkanes (C_8_–C_40_) were purchased from Fluka (Buchs, Switzerland). Phenolic acid standards, including abscisic acid (≥98%), apigenin (≥97%), apigenin-7-O-glucoside, aromadendrin, 3-hydroxytyrosol (≥98%), 4-hydroxybenzoic acid (≥99%), 4-O-caffeoylquinic acid (≥98%), caffeic acid (≥98%), dihydrocaffeic acid (98%), catechin (≥98%), chlorogenic acid, p-coumaric acid (≥98%), diosmetin, eriodictyol (≥95.0%), ethyl gallate (≥96.0%), ferulic acid, flavanomarein (≥98%), gallic acid (GA), gentisic acid (≥98%), hyperoside, hesperidin (≥80%), *p*-hydroxybenzoic acid (≥97%), 2,4-dihydroxybenzoic acid (97%), jasmonic acid, isorhamnetin (≥95.0%), luteolin (≥90%), naringin, protocatechuic acid, phloretin (≥99%), pinoresinol (≥95.0%), procyanidin B1(≥98%), rutin, salicylic acid (≥99%), sinapic acid (≥98%), syringic acid (≥95%), taxifolin, quercetin (≥95%), quinic acid, vanillin (≥97%), and vanillic acid (≥97%), were of HPLC or analytical grade and were obtained from Sigma-Aldrich (St. Louis, MO, USA) or Merck (Darmstadt, Germany).

### 3.2. Instruments

GC-MS analysis was performed using an Agilent 5975 GC-MSD system (Agilent Technologies, Santa Clara, CA, USA). UHPLC-HRMS/MS analysis was performed using a Thermo Scientific™ Vanquish™ Flex UHPLC system paired with an Orbitrap Exploris™ 120 high-resolution mass spectrometer (Thermo Fisher Scientific™, Bremen, Germany), equipped with a heated electrospray ionization (HESI) source. Absorbance measurements were conducted using a Biotek Powerwave XS microplate reader (BioTek Instruments, Winooski, VT, USA). The spectrophotometer was operated according to the manufacturer’s guidelines, and absorbance was recorded at assay-specific wavelengths. A 12-channel Eppendorf^®^ Xplorer^®^ pipette (10–300 µL) was used to transfer samples into microplate wells. A 96-deep-well round-bottom polypropylene plate (2.2 mL volume) and a 96-well flat-bottom white polystyrene microplate (non-sterile; Greiner Bio-One, Kremsmünster, Austria) were purchased from Sigma-Aldrich. For the extraction of volatile compounds, a polydimethylsiloxane/divinylbenzene (PDMS-DVB) fiber (65 µm, blue type) and a manual SPME holder (57330-U; Supelco, Bellefonte, PA, USA) were used.

### 3.3. Plant Material

Flowers, leaves, and fruits from the *M. aquifolium* plant were obtained in Günalan village, Ankara, Türkiye. During the flowering season, flowers were picked, leaves were picked along with flowers, and fruits were picked during the fruiting season. The fruit was frozen at −20 °C and then lyophilized to remove moisture. After lyophilization, the pulp and seeds of the fruit were separated manually. The seeds were properly washed to remove any remaining pulp, air-dried in the shade at ambient temperature for three days, and then stored at +4 °C until further analysis. A voucher specimen (ESSE 16356) has been prepared and deposited in the Herbarium of the Faculty of Pharmacy at Anadolu University in Türkiye.

### 3.4. Extraction Procedures

#### 3.4.1. Preparation of Lipophilic Extracts

Prior to extraction, the leaves, flowers, fruit pulp, and seeds of *M. aquifolium* were separately ground to obtain homogeneous plant materials. Lipophilic extracts were prepared by ultrasound-assisted extraction (UAE) using *n*-hexane as the extraction solvent. Approximately 40 g of powdered material from each plant organ was extracted with a total of 90 mL of *n*-hexane applied in six successive extraction cycles (15 mL per cycle). Each extraction cycle consisted of 30 min of ultrasonication in an ultrasonic water bath (40 kHz), followed by a 15 min resting period before fresh solvent was added for the subsequent extraction cycle. The extraction temperature was maintained below 40 °C throughout the procedure. The extracts obtained from all extraction cycles were combined, filtered, and concentrated under reduced pressure using a rotary evaporator, and the dried extracts were transferred into amber glass vials and stored at 4 °C until further chemical and biological analyses.

#### 3.4.2. Hydrodistillation of Essential Oil (HD)

The essential oils from air-dried flowers and leaves of *M. aquifolium* were separately extracted by hydrodistillation for 3 h using a Clevenger-type apparatus, following the procedure described in the European Pharmacopoeia [72]. The essential oil yield was calculated on a moisture-free basis. The obtained oils were dried over anhydrous sodium sulfate, transferred into amber vials, and stored at 4 °C until gas chromatography analysis.

#### 3.4.3. Microsteam Distillation–Solid Phase Microextraction (MSD-SPME)

To obtain volatile compounds extracted from the plant material, 1.0 g of leaves and flowers (separately) were put into a 25 mL flask, and 3.0 mL of distilled water was added [73]. The flask contained a Claisen distillation head and a reflux condenser, and an electric heater was used to heat the mixture. A manual SPME holder with a polydimethylsiloxane-divinylbenzene (PDMS-DVB) fiber (65 μm, blue type) was used for extraction. The fiber was conditioned at 250 °C for 15 min prior to use. The flask contained a Claisen distillation head and a reflux condenser, and an electric heater was used to heat the mixture. The MSD-SPME procedure was performed at the boiling point of water, which served as the extraction solvent. Equilibration time was defined as the period between fiber insertion and extraction initiation. After reaching equilibrium, volatile compounds were extracted for 3.0 min. Following extraction, the fiber was retracted into the needle and thermally desorbed in the injection port of the GC-FID/MS system.

#### 3.4.4. Lipid Extraction and Fatty Acid Derivatization

Fatty acid analysis followed lipid extraction and methylation by a modified biphasic partition technique according to the kit methodology [74]. The leaves, flowers, fruit pulp, and seeds of *M. aquifolium* (150 mg each) were homogenized in 3.0 mL of a chloroform:methanol combination (2:1, *v*/*v*). Phase separation commenced with the introduction of 0.5 mL of aqueous phase, succeeded by vortexing. The resulting mixture was filtered using a syringe-filter device provided by the kit. The lower organic phase, comprising chloroform and lipids, was collected. For transesterification, 200 μL of the lipid extract was evaporated under nitrogen, followed by treatment with 1.0 mL of boron trifluoride–methanol solution and 0.3 mL of *n*-hexane. The mixture was refluxed at 95 °C for 1 h to convert fatty acids into methyl esters. Following cooling, equal amounts of *n*-hexane and distilled water were incorporated. After vortexing and centrifugation (500× *g*, 5 min), the upper organic layer containing fatty acid methyl esters (FAMEs) was isolated, evaporated under nitrogen, and analyzed by GC-FID/MS.

### 3.5. Analytical Methods

#### 3.5.1. Gas Chromatography–Mass Spectrometry (GC-MS) and Flame Ionization Detection (GC-FID) Analysis

GC-FID/MS studies of essential oil volatiles, MSD-SPME extracts, and fatty acid methyl esters (FAMEs) were conducted utilizing an Agilent 7890A GC system outfitted with a 5975C MS detector and a flame ionization detector (FID) [75]. Separation was accomplished using an HP-Innowax FSC column (60 m × 0.25 mm, 0.25 µm film thickness) using helium as the carrier gas at a flow rate of 0.8 mL/min. The oven temperature was set from 60 °C (maintained for 10 min) to 220 °C at a rate of 4 °C per minute, held for 10 min, and subsequently raised to 240 °C at a rate of 1 °C per minute [76].

Volatile components (essential oils and MSD-SPME samples) were manually injected, whereas FAMEs were delivered through auto-injection. Split injection mode (40:1) was employed at 250 °C. Mass spectrometry data were acquired using electron ionization (EI) mode at 70 eV, with a mass-to-charge (*m*/*z*) ratio range of 35–450. The FID temperature was sustained at 300 °C. Compounds were identified using mass spectrum comparison with commercial libraries (Wiley-NIST, MassFinder, Adams), co-injection with known standards, and the computation of relative retention indices (RRIs) with a standard n-alkane mixture (C_8_–C_40_). Quantification was conducted utilizing FID peak regions, without normalization [77].

*M. aquifolium* leaf, flower, fruit pulp, and seed *n*-hexane extracts were silylated for GC-FID/MS chemical composition analysis. To conduct the experiment, the dry extract was dissolved in pyridine and 80 μL of BSTFA containing 1% TMCS was added. An hour was spent vortexing and heating the mixture in a 100 °C sand bath. After cooling, 1.0 μL of the silylated solution was loaded into the GC-FID/MS. An Agilent 7890A gas chromatograph and 5975C Inert Mass Selective Detector (MSD) with a triple-axis detector were used to examine *n*-hexane-extracted silylated plant compounds. Chromatographic separation took place on an HP-5 FSC column (30 m × 0.25 mm i.d., 0.25 μm film thickness; Agilent Technologies, Wilmington, DE, USA) using helium as the carrier gas at 0.8 mL/min. After increasing from 100 °C to 320 °C at 3 °C per minute, the oven was held at 320 °C for 16.67 min. Splitless injections were used. The injector and FID were heated to 250 °C and 300 °C. Mass spectra were taken at 70 eV using electron impact (EI) mode for *m*/*z* 35–1050. Compound identification was based on a comprehensive evaluation of retention times, mass spectral data, comparison with authentic reference standards, literature data, the Wiley GC/MS Library (Wiley, New York, NY, USA), and the in-house “Special Özek Silyl Derivative Library”, which has been established using authentic silylated reference compounds [78,79].

#### 3.5.2. UHPLC-HRMS/MS Orbitrap Analysis of Phenolic Compounds

Phenolic compounds in the *n*-hexane extracts of *M. aquifolium* were analyzed using a Thermo Scientific UHPLC system comprising a Dionex Ultimate 3000 RS binary pump coupled to a Q Exactive quadrupole-Orbitrap hybrid mass spectrometer (Thermo Fisher Scientific, Waltham, MA, USA) equipped with a HESI ion source. The system was operated using Xcalibur software (version 3.0.63). Chromatographic separation was carried out on an Acquity BEH C18 column (100 × 2.1 mm, 1.7 µm particle size; Waters, Milford, MA, USA) maintained at 40 °C with a flow rate of 0.5 mL/min. The mobile phase consisted of (A) water and (B) methanol, both containing 0.1% formic acid. The gradient elution program was as follows: 5% B for 1 min, increased linearly to 100% B over 9 min, maintained at 100% B for 2 min, and then returned to 5% B over 3 min. The injection volume was 5 µL. The analysis was performed in negative electrospray ionization (ESI) mode using a data-dependent acquisition (Top5) strategy. The resolution was set to 70,000 (FWHM at *m*/*z* 200) for full-scan and 17,500 for MS/MS product ion scans. HESI source parameters were as follows: spray voltage, −3.0 kV; capillary temperature, 320 °C; probe heater temperature, 400 °C; S-lens RF level, 50; sheath gas and auxiliary gas flow rates, 60 and 25 arbitrary units, respectively. Data were processed using TraceFinder 5.1 software. Identification was based on matching retention time, accurate mass (within 5 ppm), and fragment ions against a phenolic compound database containing 87 standards. Additionally, isotopic pattern scores above 80% were required for confirmation [80].

### 3.6. Total Phenol and Flavonoid Content

The Folin–Ciocalteu method, described by Singleton et al. [81], was used to measure the extracts’ total phenolic content in milligrams of gallic acid equivalents per gram. Triplicate measurements were performed (r^2^ = 0.9992).

The aluminum chloride colorimetric method was used to measure flavonoids [82]. Incubated under controlled conditions, the reaction mixture included the sample or quercetin standard, AlCl_3_, and ethanol. Absorbance measurements were recorded at 415 nm. A quercetin standard curve ranging from 0.01 to 1.0 mg/mL was employed for measurement, demonstrating exceptional linearity (r^2^ = 0.9993), with results expressed in milligrams of quercetin equivalents per gram of extract (mg REs/g_extract_). The total phenolic and flavonoid content analyses were performed in four replicates for each extract.

### 3.7. Biological Activities

#### 3.7.1. Antioxidant Activity Assays

##### Free Radical Scavenging Effect (DPPH)

The DPPH free radical scavenging activity of the extracts was assessed utilizing the modified approach by Brand-Williams et al. [83], with gallic acid used as a positive control. Stock solutions (1–10 mg/mL) were formulated in methanol with 10% DMSO. In each well of a 96-well microplate, 100 μL of the sample was combined with 100 μL of DPPH solution (0.08 mg/mL in methanol). Following a 30-min incubation at ambient temperature in darkness, absorbance was assessed at 517 nm utilizing a microplate reader. Experiments were performed in quadruplicate, and IC_50_ values were determined for samples exhibiting a minimum of 50% inhibition using SigmaPlot (v15.0). All measurements were conducted in triplicate.

##### Trolox Equivalent Antioxidant Activity (TEAC Assay)

The antioxidant capacity of the extracts was assessed using the 2,2′-Azino-bis(3-ethylbenzothiazoline-6-sulfonic acid) radical cation (ABTS^+^•) radical scavenging test, with slight changes to an established methodology [84]. ABTS radicals were generated by reacting ABTS (7 mM) with potassium persulfate and permitting the solution to incubate in the dark for 16 h. The solution was diluted with ethanol to attain an absorbance of 0.700–0.800 at 734 nm. In a 96-deep-well plate, 10 μL of each sample (extract or standard) was combined with 990 μL of ABTS^+^• solution. Following 30 min of incubation in darkness at ambient temperature, the absorbance was assessed at 734 nm utilizing a microplate reader. The antioxidant activity was quantified as Trolox equivalents (mmol TE/g_extract_), derived from the standard calibration curve: y = 24.976x + 2.6357, with r^2^ = 0.9993. All measurements were conducted in quadruplicate.

##### Oxygen Radical Absorbance Capacity (ORAC Assay)

The ORAC approach was carried out with minor alterations to an established protocol [85]. Each well of a 96-well microplate was filled with 50 μL of sample or Trolox standard (0.5–9.5 μM), 100 μL of fluorescein solution (45 nM), and 50 μL of 2,2′-Azobis(2-amidinopropane) dihydrochloride (AAPH) (15 mM). Fluorescence was recorded at 37 °C for 60 min at 1-min intervals (excitation: 485 nm; emission: 528 nm) utilizing a microplate reader (Synergy HT, BioTek Instruments, USA). The antioxidant capacity was determined by the difference in the area under the fluorescence decay curves of the sample and the blank. Results were quantified as millimoles of Trolox equivalents per milligram of extract (mmol TE/mg_extract_). All measurements were performed in quadruplicate.

##### Cupric Ion-Reducing Antioxidant Capacity (CUPRAC Assay)

The cupric ion-reducing ability of the extracts was evaluated using the CUPRAC technique [86]. Following the reaction with CuCl_2_, neocuproine, and ammonium acetate buffer, absorbance was measured at 450 nm after a 30-min incubation period. Trolox served as the calibration standard, and the results are reported as mmol TE/g_extract_. The calibration curve exhibited linearity across the examined range (y = 1.069x + 0.1341; r^2^ = 0.9986). All measurements were conducted in quadruplicate.

##### Lipid Peroxidation Inhibition Assay (β-Carotene Bleaching Test)

The antioxidant potential of the extracts was assessed utilizing a modified β-carotene bleaching technique. An emulsion was created using β-carotene, Tween-20, and linoleic acid, followed by the evaporation of chloroform and reconstitution in oxygenated water. Extracts were solubilized in methanol (with 10% dimethyl sulfoxide (DMSO)) and included in the emulsion within a 96-well plate. Absorbance was recorded at 492 nm at consistent intervals for a duration of 105 min at 50 °C. Butylated hydroxyanisole (BHA) served as the positive control. All experiments were conducted in quadruplicate. Results are presented as inhibition percentages reflecting the protection of β-carotene from oxidation [75]. All measurements were conducted in triplicate.

#### 3.7.2. α-Amylase Inhibitory Activity Assay

The α-amylase inhibitory activity of the extracts was evaluated using the iodine–potassium iodide (I_2_/KI) colorimetric method, with minor changes to previously established protocols [87]. Extracts and the conventional inhibitor, acarbose, were formulated in methanol with 10% DMSO. Reaction mixtures were formulated in 96-well microplates through the progressive incorporation of α-amylase solution (0.8 U/mL), the test or standard solution, and starch substrate. Following incubation at 37 °C, the reaction was halted with HCl, and the I_2_/KI reagent was introduced. Absorbance was quantified at 630 nm. Suitable blank and control wells were incorporated. The inhibitory activity was determined by the decrease in starch degradation relative to the control. All measurements were conducted in triplicate.

## 4. Conclusions

This study presents a comprehensive characterization of lipophilic extracts obtained from the flowers, leaves, fruit pulp, and seeds of *M. aquifolium*. Distinct variations in fatty acid distribution, volatile composition, and phenolic profiles were identified among plant organs, demonstrating the importance of organ-resolved apolar extraction strategies in revealing underexplored lipophilic constituents. Differences in phytochemical composition were reflected in variations in antioxidant and α-amylase inhibitory activities, particularly in flower and leaf extracts. These findings expand the current understanding of the lipophilic metabolome of *M. aquifolium* and provide new insights into its bioactive potential beyond the commonly studied seed oil. From a sustainable utilisation perspective, the compositional diversity observed in leaves, fruit pulp, and seeds supports their potential valorisation as sources of bioactive plant-derived ingredients. Furthermore, the application of ultrasound-assisted extraction aligns with sustainable sample preparation principles by supporting efficient recovery of lipophilic constituents through a reduced-solvent and time-efficient extraction workflow. Further studies addressing bioavailability, safety, and in vivo efficacy are necessary to substantiate practical applications.

## Figures and Tables

**Table 1:** Chemical composition of the lipophilic extracts of the leaf, flower, fruit pulp and fruit seeds of *M. aquifolium*.

RRI^#^	Compound	*n*-Hexane Extracts			
		Leaf (%)	Flower (%)	Fruit Pulps (%)	Fruit Seeds (%)
1243	Benzoic acid TMS	0.10 ± 0.00	-	-	-
1284	Phosphoric acid-3 TMS	0.19 ± 0.01	-	-	0.61 ± 0.04
1506	Malic acid-3 TMS	-	-	-	4.64 ± 0.37
1729	Levoglucosan-3 TMS	0.10 ± 0.01	-	-	-
1797	Tagatofuranose-5 TMS (isomer-2)	0.11 ± 0.01	-	-	-
1843	Neophytadiene	1.48 ± 0.00	0.33 ± 0.02	-	-
1844	α-Fructofuranose-5 TMS	-	-	-	1.45 ± 0.10
1852	β-Fructofuranose-5 TMS	-	0.10 ± 0.00	-	4.22 ± 0.34
1855	Tetradecanoic acid (=Myristic acid)-TMS	0.52 ± 0.03	-	-	-
1858	Fructopyranose-5 TMS	-	0.19 ± 0.01	-	0.53 ± 0.03
1885	β-Glucofuranose-5 TMS	-	-	-	0.55 ± 0.02
1898	Quinic acid-5 TMS	0.13 ± 0.01	0.24 ± 0.00	-	1.27 ± 0.09
1907	Lidocaine	2.38 ± 0.12	2.47 ± 0.21	-	-
1929	α-Glucopyranose-5 TMS	0.09 ± 0.00	0.22 ± 0.01	-	2.21 ± 0.15
2023	β-Glucopyranose-5 TMS	0.20 ± 0.01	0.31 ± 0.01	-	3.02 ± 0.13
2049	Hexadecanoic acid (=Palmitic acid)-TMS	2.28 ± 0.09	1.28 ± 0.06	-	3.10 ± 0.02
2127	*myo*-Inositol-6 TMS	0.11 ± 0.01	0.10 ± 0.00	-	-
2151	(*E*)-Caffeic acid-3 TMS	0.08 ± 0.00	-	-	-
2162	1-Octadecanol-TMS	0.23 ± 0.01	0.23 ± 0.01	-	-
2217	(*Z,Z*)-9,12-Octadecadienoic acid (=Linoleic acid)-TMS	0.12 ± 0.00	-	-	10.84 ± 0.21
2221	(*Z*)-9-Octadecenoic acid (=Oleic acid)-TMS	0.67 ± 0.01	0.77 ± 0.04	-	23.40 ± 1.58
2229	(*E*)-9-Octadecenoic acid (=Elaidic acid)-TMS	0.17 ± 0.00	-	-	-
2250	Octadecanoic acid (=Stearic acid)-TMS	0.44 ± 001	0.79 ± 0.01	-	0.92 ± 0.01
2448	Eicosenoic acid (=Arachidic acid)-TMS	0.31 ± 0.02	0.69 ± 0.00	-	-
2608	Monopalmitin-2 TMS	-	-	-	0.79 ± 0.05
2708	Cyclohexanedicarboxylic acid-TMS derivative	2.46 ± 0.15	-	-	-
2709	Cyclohexanedicarboxylic acid-TMS derivative	1.63 ± 0.10	1.74 ± 0.11	-	-
2713	Sucrose-8 TMS	-	-	-	12.45 ± 0.07
2716	Cyclohexanedicarboxylic acid-TMS derivative	1.49 ± 0.08	-	-	-
2734	Cyclohexanedicarboxylic acid-TMS derivative	5.12 ± 0.29	3.49 ± 0.25	-	-
2740	Cyclohexanedicarboxylic acid-TMS derivative	3.30 ± 0.18	1.53 ± 0.13	-	-
2757	Cyclohexanedicarboxylic acid-TMS derivative	5.22 ± 0.40	3.55 ± 0.03	-	-
2764	Cyclohexanedicarboxylic acid-TMS derivative	5.77 ± 0.22	3.80 ± 0.14	-	-
2778	Glyceryl monooleate-2 TMS	-	-	-	2.43 ± 0.12
2780	Cyclohexanedicarboxylic acid-TMS derivative	6.41 ± 0.35	4.23 ± 0.12	-	0.80 ± 0.02
2786	Cyclohexanedicarboxylic acid-TMS derivative	1.00 ± 0.06	-	-	-
2788	Cyclohexanedicarboxylic acid-TMS derivative	2.31 ± 0.17	1.79 ± 0.04	-	-
2800	Octacosane	-	2.67 ± 0.01	-	-
2803	Cyclohexanedicarboxylic acid-TMS derivative	3.93 ± 0.28	-	-	-
2804	1-Monostearin-2 TMS	-	-	-	0.52 ± 0.01
2808	Cyclohexanedicarboxylic acid-TMS derivative	3.48 ± 0.18	2.72 ± 0.02	-	-
2817	Cyclohexanedicarboxylic acid-TMS derivative	1.11 ± 0.01	1.01 ± 0.00	-	-
2833	Cyclohexanedicarboxylic acid-TMS derivative	4.09 ± 0.12	3.33 ± 0.11	-	-
2861	Cyclohexanedicarboxylic acid-TMS derivative	1.10 ± 0.00	0.75 ± 0.03	-	-
2900	Nonacosane	1.09 ± 0.01	1.22 ± 0.11	1.64 ± 0.08	-
2935	Catechin-5 TMS	-	-	-	-
2953	Hexacosanol-TMS	0.46 ± 0.03	-	-	-
3000	Triacontane	0.23 ± 0.01	-	0.45 ± 0.02	-
3042	Hexacosanoic acid-TMS	0.52 ± 0.03	-	-	-
3076	Nonacosan-10-ol-TMS	10.04 ± 0.01	21.10 ± 0.11	38.43 ± 0.15	1.03 ± 0.01
3100	Hentriacontane	1.38 ± 0.00	0.78 ± 0.01	2.28 ± 0.21	-
3149	Octacosan-1-ol-TMS	2.37 ± 0.12	1.10 ± 0.02	2.01 ± 0.11	-
3159	α-Tocopherol-TMS	5.51 ± 0.02	-	-	-
3244	Octacosanoic acid-TMS	0.91 ± 0.05	-	-	--
3264	5-Caffeoylquinic acid-6 TMS	-	-	2.19 ± 0.03	-
3275	Campesterol-TMS	-	-	-	1.09 ± 0.08
3347	1-Triacontanol-TMS	1.01 ± 0.01	-	0.77 ± 0.00	-
3368	β-Sitosterol-TMS	4.52 ± 0.35	3.72 ± 0.01	4.04 ± 0.18	5.37 ± 0.22
3386	Raffinose derivative	-	-	-	2.62 ± 0.15
3442	Triacontanoic acid-TMS	1.07 ± 0.08	-	0.62 ± 0.01	-
3507	Raffinose-11 TMS	-	-	-	-
3513	1-Kestose-11 TMS	-	-	-	0.45 ± 0.02
3546	1-Dotriacontanol-TMS	0.81 ± 0.02	-	0.51 ± 0.02	-
3563	Hexadecanoic acid. octadecyl ester	-	-	0.59 ± 0.03	-

RRI^#^: Relative retention index for apolar column. All values are expressed as average ± standard deviation (SD) of two independent experiments (*n* = 2).

**Table 2:** Concentration of phenolic compounds in *M. aquifolium n*-hexane extracts, as determined by UHPLC-MS/MS (mg/g_dry weight_).

Compound	*n*-Hexane Extracts			
	Flowers, mg/g_dry weight_	Leaf, mg/g_dry weight_	Fruit Pulp, mg/g_dry weight_	Fruit Seeds, mg/g_dry weight_
3-Hydroxytyrosol	2.9 ± 0.00	-	-	-
4-Hydroxybenzoic acid	0.07 ± 0.01	0.11 ± 0.00	-	15.37 ± 0.13
4-*O*-Caffeoylquinic acid	-	1.20 ± 0.05	0.003 ± 0.000	-
Caffeic acid	0.16 ± 0.00	0.29 ± 0.00	-	1.52 ± 0.07
Chlorogenic acid	61.57 ± 1.73	62.92 ± 3.31	1.57 ± 0.02	90.06 ± 1.85
Gentisic acid	-	-	-	0.44 ± 0.00
Protocatechuic Acid	0.12 ± 0.00	0.11 ± 0.01	0.62 ± 0.00	7.16 ± 0.00
Apigenin-7-*O*-Glc	-	0.26 ± 0.00	-	-
Catechin	-	-	-	4.29 ± 0.14
Diosmetin	-	0.02 ± 0.00	-	0.26 ± 0.00
Flavanomarein	-	-	-	10.87 ± 0.00
Isorhamnetin	-	-	-	12.05 ± 0.14
Luteolin	0.04 ±0.00	0.16 ± 0.00	0.14 ± 0.00	2.44 ± 0.01
Vanillin	0.60 ± 0.06	0.24 ± 0.00	0.13 ± 0.00	-

All values are expressed as average ± standard deviation (SD) of two independent experiments (*n* = 2).

**Table 3:** Biological activity of *M. aquifolium* lipophilic extracts.

Extract Type	DPPH IC_50_, mg/mL	TEAC ^(a)^ (mM TE)	CUPRAC ^(a)^ (mM TE)	ORAC ^(b)^ µmol TE/mg_extract_	β-Carotene Peroxidation Inhibition IC_50_, mg/mL	α-Amylase Inhibition IC_50_, mg/mL
Flower Extract	1.48 ± 0.13	0.020 ± 0.000	1.53 ± 0.01	0.003 ± 0.000	1.23 ± 0.00	0.16 ± 0.00
Leaf Extract	0.14 ± 0.01	0.019 ± 0.001	0.95 ± 0.01	0.452 ± 0.003	0.34 ± 0.00	0.32 ± 0.05
Fruit Pulp Extract	NE	0.021 ± 0.001	1.39 ± 0.08	0.002 ± 0.000	NE	NE
Fruit Seed Extract	0.53 ± 0.00	0.020 ± 0.000	1.71 ± 0.02	0.007 ± 0.000	0.95 ± 0.01	NE
Gallic Acid	0.003 ± 0.000	-	2.43 ± 0.14	-	-	-
BHA	-	-	-	-	0.01 ± 0.00	-
Acarbose	-	-	-	-	-	0.014 ± 0.01

BHA: Butylated hydroxyanisole; NE: Non-effective. ^(a)^ The extracts were tested in 1 mg/mL. ^(b)^: Leaf *n*-hexane extract was tested in 0.01 mg/mL; other extracts were tested in 1 mg/mL concentrations. Values are expressed as average ± SD (*n* ≥ 3). TE: Trolox equivalents.

## Data Availability

The datasets generated and analyzed during the current study are publicly available in the Zenodo repository at https://doi.org/10.5281/zenodo.20644631.

## Supplementary Materials

The supporting information is available in Zenodo at https://doi.org/10.5281/zenodo.21390910 (accessed on 9 July 2026) and includes the complete GC-FID/MS, FAME-GC, MSD-SPME, and UHPLC-HRMS/MS datasets, together with supplementary tables.
